# Hierarchical clustering of activated proteins in the PI3K and MAPK pathways in ER-positive, HER2-negative breast cancer with potential therapeutic consequences

**DOI:** 10.1038/s41416-018-0221-8

**Published:** 2018-10-05

**Authors:** Dinja T. Kruger, Karin J. Beelen, Mark Opdam, Joyce Sanders, Vincent van der Noort, Epie Boven, Sabine C. Linn

**Affiliations:** 1 Department of Medical Oncology, VU University Medical Centre, De Boelelaan 1117, 1081 HV Amsterdam, The Netherlands; 2 Division of Molecular Pathology, The Netherlands Cancer Institute, Plesmanlaan 121, 1066 CX Amsterdam, The Netherlands; 3 Department of Medical Oncology, Reinier de Graaf Groep, Reinier de Graafweg 5, 2625 AD Delft, The Netherlands; 4 Department of Pathology, The Netherlands Cancer Institute, Plesmanlaan 121, 1066 CX Amsterdam, The Netherlands; 5 Division of Biometrics, The Netherlands Cancer Institute, Plesmanlaan 121, 1066 CX Amsterdam, The Netherlands; 6 Department of Medical Oncology, The Netherlands Cancer Institute, Plesmanlaan 121, 1066 CX Amsterdam, The Netherlands; 7 Department of Pathology, University Medical Centre Utrecht, and Utrecht University, Heidelberglaan 100, 3584 CX Utrecht, The Netherlands

**Keywords:** Predictive markers, Breast cancer, Breast cancer, Prognostic markers

## Abstract

**Background:**

The phosphatidylinositol-3-kinase (PI3K) and/or mitogen-activated protein kinase (MAPK) pathways are frequently activated in breast cancer which can result in antioestrogen resistance. Single protein markers failed to be introduced into clinical practice. We, therefore, aimed to find a better read-out of activation of the PI3K and MAPK pathways in ER+/HER2− breast cancer. Assessment of seven PI3K/MAPK proteins might better reflect pathway activation and distinguish patients without adjuvant tamoxifen benefit.

**Methods:**

Tumour blocks were recollected from 293 primary postmenopausal ER+/HER2− breast cancer patients randomised between tamoxifen and no adjuvant therapy. PTEN, p-AKT(Thr308), p-AKT(Ser473), p-p70S6K, p-4EBP1, p-ERK1/2 and p-S6RP expression was assessed by immunohistochemistry followed by unsupervised hierarchical clustering. The primary endpoint was recurrence-free interval. Multivariate Cox models were used to assess tamoxifen benefit. A classification tool was developed based on protein expression profile.

**Results:**

Subgroups were identified with preferentially activated (A) and preferentially not activated (N) proteins. Patients in group *N* derived significant benefit from tamoxifen (multivariate hazard ratio (HR) = 0.23, *p* = 0.000101), while patients from group A did not (multivariate HR = 1.37, *p* = 0.64), *p* for interaction 0.020. Our generated classification tool confirmed these results (*p* for interaction 0.024).

**Conclusions:**

Hierarchical clustering of seven PI3K/MAPK proteins reflects pathway activation and can guide treatment decisions in primary ER+/HER2− postmenopausal breast cancer patients.

## Background

Both the phosphatidylinositol-3-kinase (PI3K)/AKT/mammalian target of rapamycin (mTOR), and the mitogen-activated protein kinase (MAPK) pathways are important in normal cell function and cancer cell biology. By the transmission of cell signals in response to extracellular events, they promote RNA translation, proliferation, growth and cell survival (Fig. S1).^1,2^ In breast cancer, cross-talk between both pathways and the oestrogen receptor (ER) signalling pathway has been described.^2–5^

The PI3K pathway is the most frequently altered pathway in breast cancer.^5,6^ Alterations in genes encoding proteins in this pathway occur in over 75% of primary breast cancer cases,^6^ but the numbers vary among breast cancer subtypes. For example, activating mutations in *PIK3CA*, which encodes the catalytic subunit of PI3K, occur most frequently in ER-positive (29–45%) and human epidermal growth factor receptor 2 (HER2)-positive tumours (39%), and less in triple-negative breast cancer (TNBC) cases (9%).^7^ Alterations in protein expression of the tumour regulator PTEN are most often seen in TNBC (35–67%) compared to 29–44% in ER-positive breast tumours and 19–22% of HER2-positive breast cancer cases.^7^ High levels of phosphorylated Akt are common in all breast cancer subtypes of which p-Akt(Ser473) has most often been studied.^8^ Overexpression of p-Akt(Ser473) can be detected in 78% of luminal-like breast cancer, in 80% of HER2-positive breast cancer and in 58–62% of TNBC cases.^9^

The MAPK pathway is less frequently altered in breast cancer. Mutations in genes encoding this pathway range from 2 to 10%.^10^ Activation seems most important in the tumour biology of TNBC.^1^ Umemura et al.^11^ have demonstrated more phosphorylation of ERK1/2 in TNBC than in other subtypes, although this result was not significant possibly due to the small sample size. Analysis of an ERK1/2 miRNA signature has indicated that a high activation status was significantly associated with ER-negativity, high tumour grade, increased proliferation, basal and HER2 molecular subtypes and poor clinical outcomes.^12^

Increasing evidence exists that activation of the PI3K pathway or the MAPK pathway in breast cancer is associated with a higher chance to develop resistance to anti-oestrogens or oestrogen deprivation.^4,13,14^ Pathway activation may also influence prognosis.^8,15–17^ Therefore, research has focused on the identification of predictive and prognostic biomarkers associated with pathway activation that might be relevant for patient selection. Consequently, both total levels and phosphorylation of proteins downstream in the PI3K and MAPK pathways have been studied. Phosphorylated proteins, however, are considered as typical markers for pathway activation.

Previously, our group has demonstrated that high levels of phosphorylated p70S6K (p-p70S6K) in primary breast cancer were associated with less adjuvant tamoxifen benefit.^18^ Similarly, tamoxifen was less effective in patients with tumours that expressed high p-mTOR and positive p-ERK1/2.^18^ Analysis of the tumour material for changes in the single proteins PTEN, p-AKT(Ser473), p-AKT(Thr308), p-mTOR, p-ERK1/2 as well as the presence of *PIK3CA* mutations and a possible association with tamoxifen benefit was negative.^18,19^ The phosphorylation status of two other proteins downstream in the PI3K/MAPK pathways, p-4EBP1 and p-S6RP, is often used as a measure of activation of the PI3K pathway.^20^ These proteins act separately downstream of mTOR (Figure S1) and might also serve as markers for endocrine therapy benefit. We now analysed the predictive and prognostic potential of p-4EBP1 and p-S6RP expression in the same primary breast cancer samples as examined before.^18,19^

Since various feedback loops and cross-talks between the pathways exist, a single marker is at risk to produce false positive- or false-negative results when used as readout for pathway activation. Therefore, the aim of this study was to search for a better read-out of activated proteins of the PI3K and MAPK pathways in ER-positive (ER+)/HER2-negative (HER2–) breast cancer patients, predictive for endocrine resistance or associated with prognosis. Unsupervised hierarchical clustering was performed to show for the first time how seven proteins downstream in both pathways are expressed in ER+/HER2– breast cancer cases. We assessed the prognostic value of panels of protein expression levels reflecting differences in activation status of the PI3K/MAPK pathways in ER+/HER2– breast cancer patients as well as of a potential tool to predict the usefulness of adjuvant tamoxifen.

## Methods

### Patients and material

Primary tumour tissue blocks were recollected from postmenopausal patients with stage I–III breast cancer who participated in the IKA trial and were randomised between adjuvant tamoxifen vs. no adjuvant endocrine therapy.^21^ The patient characteristics and clinical outcome of the original study population have been presented elsewhere^18,21^, and are part of the Oxford patient-level meta-analysis.^22^ A summary of the IKA trial can be found in the Supplemental methods.

Tumour material was available from 739 patients. These patients did not differ in clinico-pathological characteristics from the total group (Table 1).^18,19^ From the formalin-fixed paraffin-embedded tumour blocks, tissue microarrays (TMAs) were constructed using three 0.6 mm cores. Each TMA was stained for ERα, progesterone receptor (PR), HER2 and Ki67 (Supplementary methods). The mitotic count was assessed per 2 mm^2^ as before.^23^ Tumour grade was scored on a haematoxylin–eosin stained slide according to the modified Bloom and Richardson scoring system.^24^ For this retrospective translational study, no additional consent was required according to Dutch legislation,^25^ since the use of anonymised archival pathology left-over material does not interfere with patient care. Tumour tissue was handled according to the Dutch code of conduct for responsible use of human tissue in the context of health research.^26^

**Table 1:** Distribution of clinico-pathological characteristics of patients presented in the ER-positive, HER2-negative heatmap and subgroups (A and N) as well as in the original data set of patients with tumour material available and in the total IKA trial population

	Heatmap group A	Heatmap group N	Total heatmap population	Total ER+/HER2− population	Patients with tumour material available	Total IKA study population
	*n* (%)	*n* (%)	*n* (%)	*n* (%)	*n* (%)	*n* (%)
Total	111 (38)	182 (62)	293 (100)	489 (100)	739 (100)	1662 (100)
*Age*						
<65	59 (53)	84 (46)	143 (49)	233 (48)	378 (51)	869 (52)
≥65	52 (47)	98 (54)	150 (51)	256 (52)	361 (49)	793 (48)
*Lymph node status*						
Negative	64 (58)	95 (52)	159 (54)	276 (56)	393 (53)	901 (54)
Positive	47 (42)	87 (48)	134 (46)	213 (43)	346 (47)	761 (46)
*T stage*						
T1–2	101 (91)	160 (88)	261 (89)	437 (89)	659 (89)	1482 (89)
T3–4	10 (9)	22 (12)	32 (11)	52 (11)	80 (11)	180 (11)
*Grade*						
Grade 1–2	72 (65)	114 (63)	186 (63)	342 (70)	435 (59)	435 (59)^a^
Grade 3	39 (35)	68 (37)	107 (37)	147 (30)	304 (41)	304 (41)^a^
*Histological subtype*						
Ductal	79 (93)	154 (94)	233 (94)	347 (87)	540 (89)	540 (89)^a^
Lobular	6 (7)	10 (6)	16 (6)	50 (13)	66 (11)	66 (11)^a^
*HER2 status*						
Negative	111 (100)	182 (100)	293 (100)	489 (100)	594 (88)	594 (88)^a^
Positive	0 (0)	0 (0)	0 (0)	0 (0)	85 (12)	85 (12)^a^
*PR status*						
Negative	44 (40)	84 (46)	128 (44)	222 (46)	414 (57)	346 (40)^b^
Positive	67 (60)	97 (54)	164 (56)	261 (54)	304 (43)	513 (60)^b^
*ER status*						
Negative	0 (0)	0 (0)	0 (0)	0 (0)	159 (23)	311 (23)^c^
Positive	111 (100)	182 (100)	293 (100)	489 (100)	563 (77)	1014 (77)^c^

^a^Only revised scorings from 739 patients of the IKA trial population from whom tumour tissue could be obtained are shown ^b^Determined by progesterone receptor (PR) ligand binding assay in original trial, missing data of 803 patients ^c^Determined by oestrogen receptor (ER) ligand binding assay in original trial, missing data of 337 patients

### Immunohistochemistry

TMAs have previously been analysed for PTEN, p-AKT(Thr308), p-AKT(Ser473), p-p70S6K and p-ERK1/2 of which methodology for scoring is summarised in Table S1.^18,19^ The age of the tumour samples and different fixation procedures did not affect the phospho-protein staining procedure.^18,19^ For p-4EBP1 and p-S6RP, staining was performed using a standardised protocol on the Ventana Benchmark^®^ Ultra system (Ventana Medical Systems, Tucson, Arizona, USA). To ensure phospho-specificity for all phospho-antibodies, a test TMA was treated with λ-phosphatase before staining, resulting in disappearance of the positive staining. For p-4EBP1, the percentage of tumour cells with positive nuclear staining was scored. For p-S6RP, the percentage of tumour cells with positive cytoplasmic and membranous staining was scored. Similar as before, the highest score out of three cores from the same tumour tissue block was used for the statistical analyses. The inter observer variability was determined by independent assessment of one stained TMA for each antibody by a second blinded observer. To that end, the scorings were analysed as binary factor using the median as cut-off to calculate the inter-observer variability expressed as kappa coefficient (Table S1).^27^ For further analyses we used the scores generated by one of the two observers (M.O.).

### Hierarchical clustering

Hierarchical clustering was performed using the continuous scoring values of all seven proteins to create a heatmap. The dendrogram of the heatmap was created by unsupervised clustering, meaning that the clusters formed were based purely on the similarities and dissimilarities among the patients by the expressions of the seven proteins. No information about the patients’ survival or any other information about the patient or arm of randomisation was used generating the heatmap. Concretely, the dendrogram was computed as follows. In the subset of 293 ER+/HER2− tumours for which scorings of the seven proteins of interest were available, we first normalised the scores by dividing each number by the standard deviation of the scores for that protein. On the resulting rescaled dataset, hierarchical clustering was carried out by the “heatmap.2” function of the R-package “gplot”, using the Euclidean distance to determine the similarity among the tumours and a complete linkage function to iteratively build up the clusters. These latter two choices are the default settings in R’s “heatmap.2” function. The heatmap cluster showing the highest levels of activation of the proteins was denoted subgroup A and the cluster showing less activation was denoted subgroup N.

### Classification tool

A practical disadvantage of hierarchical clustering is that it cannot be used to classify a new patient into one of the two clusters. As a solution, we devised a classification tool for ER+/HER2− patients. With this rule, a new patient would be assigned to either the A or the N subgroup based on a particular expression profile of the seven proteins. The classification tool was optimised for reproducing the clusters detected by unsupervised hierarchical clustering without information on treatment or outcome.

### Statistics

Recurrence-free interval (RFI) was defined as the time from the first randomisation to the occurrence of a local, regional or distant recurrence or breast cancer-specific death.^28^ Patients with a secondary contralateral breast tumour were censored at the time of the contralateral diagnosis, since it was not possible to link breast cancer-specific events to the first or to the contralateral malignancy. Patients who died from other causes or who were lost to follow up, for instance because follow up was no longer required according to local practice, due to emigration or by patient’s request, were censored at the time of this occurrence. The Kaplan–Meier method was used to construct survival curves. The primary end-point of all analyses, tamoxifen benefit, was defined as the HR for RFI (as estimated by a Cox proportional hazard model) between the two arms of randomisation: adjuvant tamoxifen for 1–3 years (TAM) and no adjuvant treatment (CON).

p-4EBP1 and p-S6RP were dichotomised before entering further analyses. An exploratory analysis to test which binary score would give the best prediction of tamoxifen benefit with p-4EBP1 and p-S6RP was performed by using multiple cut-off points for p-4EBP1 and p-S6RP. The binary score which reached the lowest *p* value for interaction (i.e., showing the clearest difference in tamoxifen benefit between the two resulting subgroups) was used in further analyses. Fisher’s exact test was used to test the association between expression of p-4EBP1, p-S6RP and clinico-pathological characteristics or the other proteins associated with activation of the PI3K/MAPK pathway.

Tamoxifen benefit of high vs. low p-4EBP1, high vs. low p-S6RP, subgroup A vs. N identified by hierarchical clustering, and subgroup FALSE and TRUE identified by our classification tool was estimated in bivariate and multivariate Cox proportional hazard regression analyses with a term for interaction between arm of randomisation and the variable of interest. Known prognostic variables were included in the multivariate analyses as covariates: age (<65 vs. ≥65), histological grade (grade 3 vs. grade 1–2), tumour size (T3–T4 vs. T1–T2), histological subtype (lobular vs. ductal), PR status (negative vs. positive), Ki67 score (<10% vs. ≥10%) and mitotic count (<8 mitotic counts/2 mm^2^ vs. ≥8 mitotic counts/2 mm^2^). All Cox models were stratified for nodal status.

To test whether p-4EBP1 or p-S6RP expression levels, heatmap or classification tool subgroups were associated with prognosis of ER+ patients, univariate and multivariate Cox proportional hazard regression analyses were performed and stratified for lymph node status. Covariates included in the multivariate tests were treatment arm, age (<65 vs. ≥65), histological grade (grade 3 vs. grade 1–2), tumour size (T3–T4 vs. T1–T2), histological subtype (lobular vs. ductal), PR status (negative vs. positive), Ki67 score (<10% vs. ≥10%) and mitotic count (<8 mitotic counts per 2 mm^2^ vs. ≥8 mitotic counts per 2 mm^2^). Prognostic analyses were carried out both in the entire study cohort using treatment arm as a covariate, as well as in the tamoxifen and control arm separately.

This study complied with reporting recommendations for tumour marker prognostic studies (REMARK) criteria.^29^ The analyses and heatmaps were generated using R for statistics (Windows version 3.3.1).

## Results

### Hierarchical clustering of proteins associated with the PI3K and MAPK pathways and differences in tamoxifen benefit and/or prognosis

The results regarding p-4EBP1 and p-S6RP as single markers can be found in the Supplemental material (Results, Table S2 and Figure S2). In summary, neither p-4EBP1 nor p-S6RP appeared to have prognostic value or were useful to predict tamoxifen failure. We, therefore, hypothesised that discriminating tumours differing in general activation of the PI3K/MAPK pathways based on seven downstream protein expression levels would enable identifying patients without tamoxifen benefit.

**Fig. 1:**
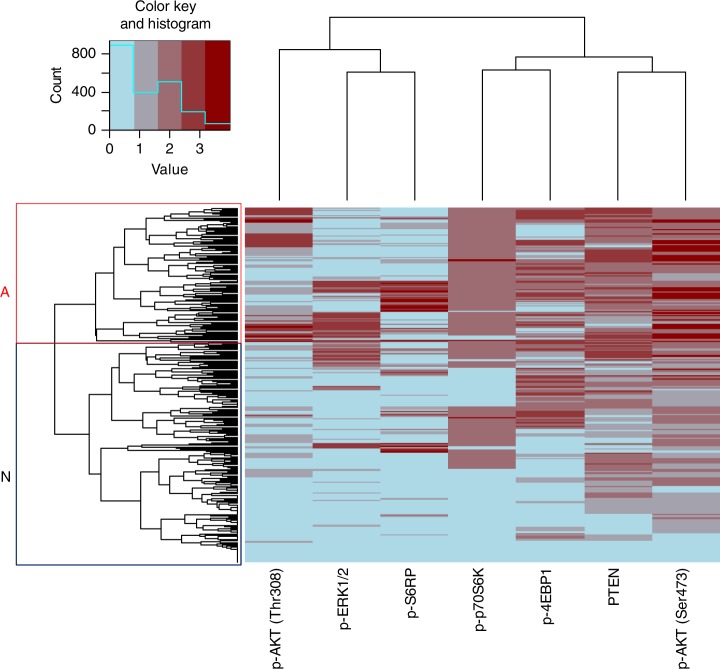
Hierarchical clustering of seven proteins in ER-positive, HER2-negative tumours visualised in a heatmap. Red bars indicate a higher score in activation. Blue bars indicate a lower score and, therefore, less activation of the corresponding protein. The red box classifies the patients in group A with a more activated PI3K and/or MAPK pathway, while the black box identifies patients in group N in whom these pathways are grossly not activated. p-ERK1/2 phosphorylated extracellular signal-regulated kinase 1 and 2; p-S6RP phosphorylated 40S ribosomal protein S6; p-p70S6K phosphorylated p70 ribosomal protein S6 kinase; p-AKT phosphorylated AKT at phospho-sites Thr308 and Ser473; p-4EBP1 initiation factor eukaryotic initiation factor 4E binding protein 1; PTEN phosphatase and tensin homologue

**Fig. 2:**
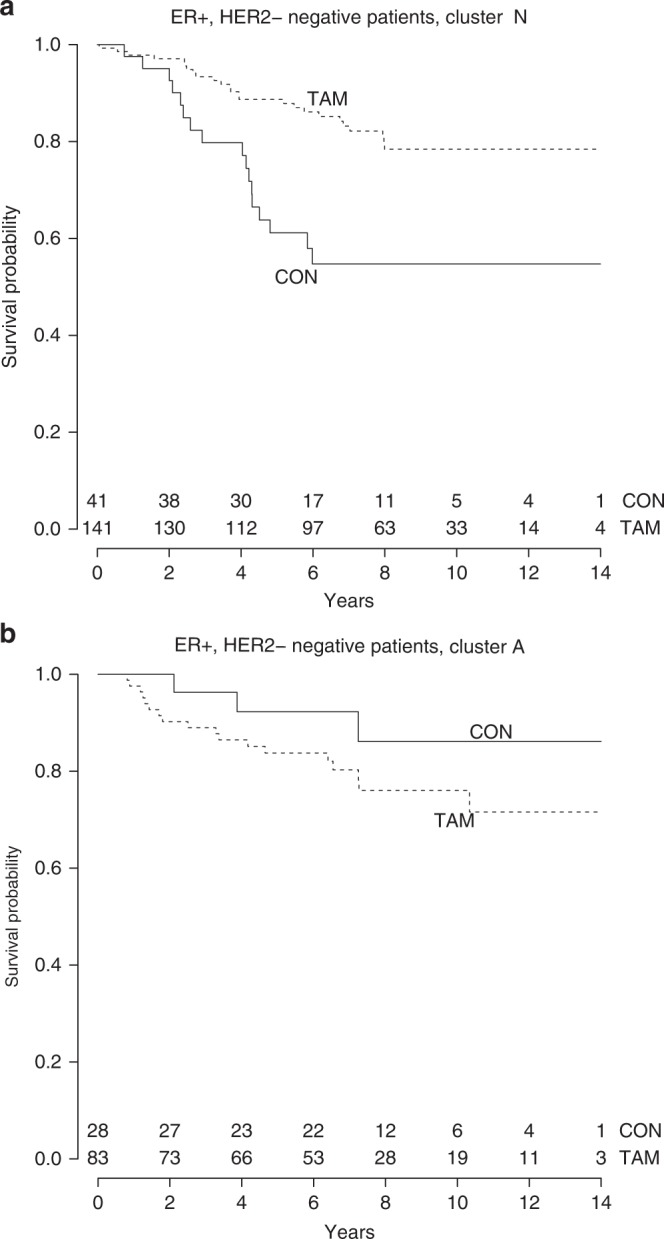
Kaplan–Meier analyses for recurrence-free interval in ER-positive, HER2-negative breast cancer patients according to tamoxifen treatment and (**a**) hierarchical cluster N, indicating patients assigned to group N (pathways grossly not activated) and (**b**) hierarchical cluster A, indicating patients assigned to group A (preferentially activated PI3K and/or MAPK pathway). The continuous line shows patients randomised to the control arm

A total of 489 tumours were ER+/HER2– and of these, 293 samples had with scorings for all seven proteins. In this cohort, a total of 63 RFI events were observed. The median follow-up of patients without a recurrence event was 8.2 years (95% CI: 7.6–8.7). Unsupervised hierarchical clustering of the proteins in the PI3K/MAPK pathways yielded two patient subgroups (A: preferentially activated and N: preferentially not activated) (Fig. 1). There were no significant differences in clinico-pathological characteristics between the two heatmap subgroups (Tables 1 and 2). More proteins were activated in tumour tissue from patients in group A, while in tumours of patients from group N activation was relatively absent. Patients in group N derived significant benefit from tamoxifen (multivariate HR = 0.23, 95% CI: 0.11–0.49, *p* = 0.000101) (Tables 3 and S3A), while patients in group A did not (multivariate HR = 1.37, 95% CI: 0.38–4.97, *p* = 0.636) (Tables 3 and S3A). Figure 2 shows the Kaplan–Meier curves for the heatmap subgroups by treatment arm. When stratified for lymph node status, a significant interaction for heatmap group with tamoxifen efficacy was observed (multivariate *p* for interaction = 0.020).

**Table 2:** Association between clinico-pathological factors in heatmap cluster A vs. N and classification tool TRUE vs. FALSE (*n* (%))

	Heatmap (*n* = 293)		*p* Value*	Classification tool (*n* = 293)		*p* Value*
	A	*N*		TRUE	FALSE	
	111 (38)	182 (62)		124 (42)	169 (58)	
*Age*						
<65	59 (53)	84 (46)	0.28	63 (51)	80 (47)	0.64
≥65	52 (75)	98 (54)		61 (49)	89 (53)	
*Lymph node status*						
Negative	64 (58)	95 (52)	0.4	73 (59)	86 (51)	0.19
Positive	47 (42)	87 (48)		51 (41)	83 (49)	
*T stage*						
T1–2	101 (91)	160 (88)	0.45	111 (90)	150 (89)	1
T3–4	10 (9)	22 (12)		13 (10)	19 (11)	
*Grade*						
Grade 1–2	72 (65)	114 (63)	0.71	82 (66)	104 (62)	0.46
Grade 3	39 (35)	68 (37)		42 (34)	65 (38)	
*Histological subtype*						
Ductal	79 (93)	154 (94)	0.79	88 (94)	145 (94)	1
Lobular	6 (7)	10 (6)		6 (6)	10 (6)	
*PR status*						
Negative	44 (40)	84 (46)	0.28	50 (40)	78 (46)	0.34
Positive	67 (60)	97 (54)		74 (60)	90 (54)	
*Ki67*						
<10%	66 (60)	159 (88)	<0.0001	77 (63)	148 (88)	<0.0001
≥10%	44 (40)	22 (12)		46 (37)	20 (12)	
*Mitotic count*						
<8/2 mm²	51 (46)	84 (47)	1	55 (45)	80 (48)	0.72
≥8/2 mm²	59 (54)	96 (53)		67 (55)	88 (52)

**Table 3:** Multivariate Cox proportional hazard model of recurrence free interval (RFI) including heatmap group and treatment interaction

Variable		HR	95% CI	*p*
Interaction	Cluster vs. treatment			0.012
Tamoxifen vs. CON (ref)	In cluster N	0.257	0.125–0.529	0.00023
	In cluster A	1.670	0.469–5.943	0.429
Cluster A vs. N (ref)	CON patients	0.229	0.061–0.856	0.0283
	TAM patients	1.49	0.74–3.00	0.261
Age	≥65 vs. <65 (ref)	1.091	0.630–1.890	0.757
T stage	T3–4 vs. T1–2 (ref)	1.965	0.977–3.951	0.058
Grade	Grade 3 vs. grade 1–2 (ref)	1.813	0.805–4.080	0.151
PR status	Positive vs. negative (ref)	1.489	0.850–2.606	0.164
Histological subtype	Lobular vs. ductal (ref)	2.630	0.966–7.156	0.058
Ki67	≥10% vs. <10% (ref)	1.258	0.619–2.556	0.525
Mitotic count	≥8/2 mm² vs. <8/2 mm² (ref)	0.734	0.316–1.703	0.471

The prognostic potential of heatmap cluster was analysed only in patients randomised to the control arm to rule out bias from treatment. Interestingly, control patients in group A demonstrated a better prognosis than control patients in group N when stratified for lymph node status (multivariate HR = 0.061, 95% CI: 0.0079–0.47, *p* = 0.0073) (Table S3B) as shown in Fig. 2 (CON groups).

### Classification tool

**Fig. 3:**
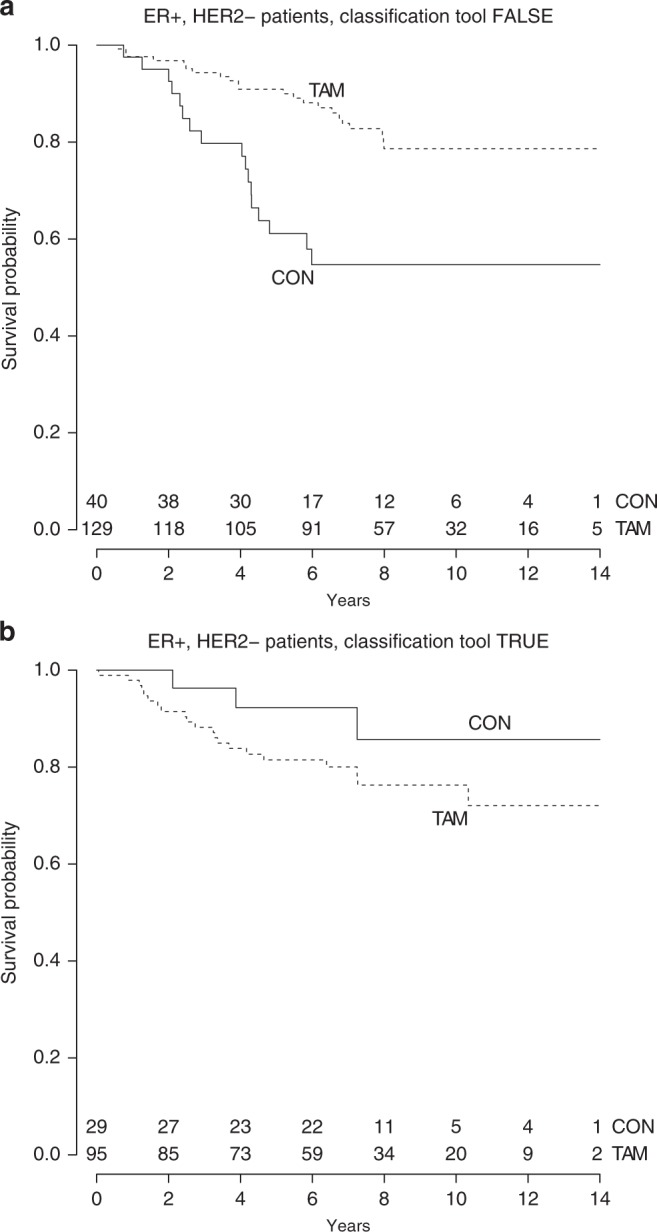
Kaplan–Meier analyses for recurrence-free interval in ER-positive, HER2-negative breast cancer patients according to tamoxifen treatment and (**a**) patients assigned to group FALSE (corresponding to the preferentially not activated heatmap group N) by use of our classification tool; and (**b**) patients assigned to group TRUE (corresponding to the preferentially activated heatmap group A) by use of our classification tool. The interrupted line specifies patients treated with tamoxifen. The continuous line shows patients randomised to the control arm

A decision rule was developed based on the expression profile of the seven proteins by hierarchical clustering, blinded to outcome, to classify future postmenopausal patients with primary ER+/HER2– breast cancer into one of the heatmap subgroups. When a patient fulfilled at least one of the following four criteria, the rule would be TRUE and the patient would be assigned to the activated A group: [p-AKT(Thr308) > 1]; or [p-S6RP > 70%]; or [p-4EBP1 > 50% and p-ERK1/2 > 70%]; or [p-p70S6K > 0 and PTEN > 1 and p-AKT(Ser437) > 1]. If none of these criteria was met, the rule would be FALSE and the corresponding patient would be categorised in the not activated N group. After application of this classification tool in the 293 ER+/HER2– patients, 88% were categorised in the same heatmap subgroup as observed with unsupervised hierarchical clustering. The clinico-pathological characteristics of the classification tool subgroups are shown in Table 2. The classification tool was equally successful in differentiating ER+/HER2– patients with or without tamoxifen benefit (*p* for interaction = 0.024). When the rule was TRUE, no benefit of tamoxifen was observed (multivariate HR = 1.30, 95% CI: 0.36−4.68, *p* = 0.69) (Table S3C, D). When the rule was FALSE, patients derived significant benefit from tamoxifen (multivariate HR = 0.25, 95% CI: 0.07−0.91, *p* = 0.000124) (Table S3C,D). The Kaplan–Meier curves are visualised in Fig. 3.

Similar to the heatmap cluster analyses, the prognostic potential of the classification tool was analysed only in patients randomised to the control arm and stratified for lymph node status. When the rule was TRUE, control patients demonstrated a better prognosis than when the rule was FALSE (multivariate HR = 0.065, 95% CI: 0.0091−0.469, *p* = 0.0067) (Table S3E) as shown in Fig. 3 (CON groups).

## Discussion

We demonstrate by unsupervised hierarchical clustering of seven proteins that ER+/HER2– breast cancer patients with a tumour containing preferentially activated PI3K/MAPK pathways derived no benefit from adjuvant tamoxifen, while those without preferential activation had an improved RFI on tamoxifen. Our classification tool successfully categorised patients in these two subgroups based on their immunohistochemistry scorings. Further, patients in the more activated PI3K/MAPK pathway group had a better prognosis.

Within the ER+/HER2– breast cancer samples, we show for the first time that unsupervised hierarchical clustering resulted in the distinction of tumours with a preferentially activated (group A) and not activated (group N) profile categorising patients with differential adjuvant tamoxifen benefit. Further, we successfully generated a tool to classify future ER+/HER2– breast cancer patients in subgroups A or N based on particular protein expression profiles. The clustering method has been used before by Horii et al.^17^ to explore biomarkers for clinico-pathological relevance. They examined 337 unselected primary breast cancer cases for staining scores of p-AKT(Ser473), cyclin D1, P27, p-p70S6K, p-4EBP1 and p-ERK1/2, and cluster classification showed significant relationships with subgroups expressed by hormone receptor, HER2, grade and histological subtype, but not with prognosis. Their study design did not allow for insight in treatment benefit.

Activation of the PI3K/MAPK pathways in the ER+/HER2– breast cancer samples was correlated with a better prognosis. Although this might seem a counterintuitive finding, it is in line with previous research. We have previously shown that patients with ER+ tumours harbouring high p-AKT(Ser473), p-AKT(Thr308), p-mTOR or p-p70S6K expression who did not receive adjuvant tamoxifen also had a decreased risk of breast cancer recurrence.^18^ Other groups have analysed the prognostic significance of *PIK3CA* mutations or the expression of individual proteins. In a recent meta-analysis,^30^ *PIK3CA* mutation status was not associated with relapse-free or overall survival in hormone receptor-positive breast cancer patients. Bostner et al.^31^ have not detected prognostic value for either p-mTOR or p-Akt(Ser473), but high expression of S6K1 predicted a worse prognosis.^32^ In the same cohort of patients, Karlsson et al.^33^ have described that strong nuclear 4EBP1 correlated with good prognosis, while strong cytoplasmic p-4EBP1 was a poor prognostic factor. In all, future research should reveal whether a panel of (phosphorylated) proteins related to the PI3K/MAPK pathways in hormone receptor-positive breast cancer might better reflect the true activation status of these pathways, and therefore give more precise prognostic information than single markers. For instance, earlier clinical research already demonstrated the lack of correlation between the presence of *PIK3CA* mutations and the protein activation status of these same pathways.^6,19^ Furthermore, considering it is unclear how an activated PI3K pathway can provide a better prognosis, it would be very useful if this becomes the subject of future research as well.

High levels of cytoplasmic PTEN were positively associated with PI3K pathway activation in our patient cohort. *PTEN* is generally known as a tumour suppressor gene and inactivation has been shown to be involved in heritable and sporadic cancer types.^34^ The incidence of mutations or deletions in *PTEN* is low in primary tumours,^34^ being 4% in primary breast cancer.^6^ PTEN staining results were only found to be weakly correlated with *PTEN* gene expression.^35^ Localisation of PTEN appears to be cell cycle dependent; quiescent tissues exhibit predominantly nuclear localisation, whereas cancerous tissues have a higher percentage of cells in S phase and exhibit increased levels of cytoplasmic PTEN.^34^ Cytoplasmic PTEN has several proteoforms varying in functionality.^36^ Tumours with a high degree of immunoreactivity with a PTEN antibody may in fact reflect functionally PTEN-deficient samples.^36^ These findings could be an explanation that cytoplasmic PTEN expression is positively associated with PI3K pathway activation in our study.

Whether our classification tool can be used to distinguish high-risk postmenopausal patients with primary ER+/HER2– breast cancer that do not benefit from adjuvant tamoxifen should be validated in a retrospective independent trial cohort before it can be implemented in the clinic. The strength of the IKA trial of which we recollected the tumour tissue from the participating patients, is that it included a control group not receiving any systemic treatment to discern a predictive from a prognostic effect of a biomarker.^37^ This kind of retrospective cohorts are scarce and it would not be ethically justified to withhold a control group adjuvant hormonal therapy today when setting up a new trial. Two retrospective trials that could be used to validate our results are the Stockholm trial^32,33^ and/or the National Surgical Adjuvant Breast and Bowel Project (NSABP) B-14 trial.^38^ Before using such valuable patient material, more evidence should be generated on less precious material. For this, a matched case-control study could be set up using the Netherlands Cancer Registry database coupled with the nationwide digital pathology archive, through which tumour material can be recollected for research purposes, e.g., the PARADIGM initiative.^39^

Current adjuvant endocrine therapy in postmenopausal patients generally consists of tamoxifen given in sequence with an aromatase inhibitor (AI) for at least 5 years.^40^ In our study, patients received only 1–3 years of tamoxifen without the addition of an AI. Since PI3K pathway activation is associated with endocrine resistance irrespective of the given treatment,^5^ we anticipate that our findings might also be applicable to indicate a subgroup of patients without advantage from adjuvant AIs. Although we have no information on the meaning of our results when applied to longer treatment duration with tamoxifen, we think that it is highly unlikely that a tumour with intrinsic resistance against tamoxifen via activated PI3K/MAPK pathways, will lose this resistance mechanism after longer treatment with the same drug. We, therefore, assume that our findings can also be applied to patients who receive current standard care and this could in the future be addressed by analysing studies like the NSABP B-14 trial.^38^ For premenopausal patients, the most recent ASCO guideline recommends offering adjuvant tamoxifen for at least 5 or even 10 years.^40^ We demonstrated that the absence of activated PI3K/MAPK pathways in the primary tumour was predictive for substantial tamoxifen benefit in postmenopausal patients. However, whether these findings will also be applicable in the premenopausal setting is currently unknown and requires further research.

In our study a substantial proportion of patients had a PI3K/MAPK pathway activation (111/293 = 38%) and did not derive benefit from adjuvant tamoxifen. Nevertheless, not all PI3K/MAPK pathway-negative patients continued to be recurrence-free after tamoxifen, indicating that additional resistance mechanisms remain to be identified.^41^

Currently, a number of multigene tests are available, such as the MammaPrint, PAM50-based risk of recurrence, Oncotype DX, IHC4 score and Breast Cancer Index.^42,43^ These tests have been investigated for the prediction of outcome after endocrine treatment and/or endocrine therapy benefit in ER+/HER2− breast cancer patients. Although they added valuable prognostic information, the most recent ASCO guidelines recommend using them for clinical decisions on adjuvant chemotherapy only and not for decisions regarding adjuvant endocrine therapy due to lack of evidence for that specific goal.^42,43^ To decide whether or not adjuvant endocrine (mono) therapy should be used, our classification tool could be of added value.

In conclusion, our study demonstrates the value of determining the expression of multiple proteins as readout for PI3K/MAPK pathway activation to predict adjuvant tamoxifen benefit in an ER+/HER2– breast cancer cohort. Furthermore, we developed a tool to classify patients into two categories that either derive substantial benefit or not from adjuvant tamoxifen on the basis of seven proteins at predefined cut-off expression levels. Before introduction into daily clinical practice, these results require validation in an independent study.

## Electronic supplementary material

Supplementary Information

## **Availability of data and material**

The data that support the findings of this study are available from the corresponding author upon reasonable request.
