# Supplementary material for: Hierarchical clustering of activated proteins in the PI3K and MAPK pathways in ER-positive, HER2-negative breast cancer with potential therapeutic consequences
Source: Br J Cancer. 2018 Oct 5;119(7):832–9. doi: 10.1038/s41416-018-0221-8 (PMC6189147; doi:10.1038/s41416-018-0221-8)
Supplement: Supplementary file 1 — Supplementary Information [file 41416_2018_221_MOESM1_ESM.docx]

## Supplemental Material to

# Hierarchical clustering of activated proteins in the PI3K and MAPK pathways in ER-positive, HER2-negative breast cancer with potential therapeutic consequences

Dinja T Kruger, Karin J Beelen, Mark Opdam, Joyce Sanders, Vincent van der Noort, Epie Boven, Sabine C Linn

## Supplementary Methods

*Patients and material*

The IKA trial (July 1982 until September 1993) included postmenopausal patients with stage I-III breast cancer who were randomised in a 2:1 ratio between adjuvant tamoxifen (30 mg/day) for 1 year versus no adjuvant endocrine therapy. After 1 year, patients on tamoxifen were randomised a second time to continue another 2 years of tamoxifen or to stop further treatment. In 1988, based on two interim analyses demonstrating a significant improvement in recurrence-free survival (RFS) in lymph node-positive patients, all node-positive patients were given 1 year of tamoxifen before they participated in the second randomization. None of the patients received chemotherapy. The IKA trial was approved by the central ethics committee of the Netherlands Cancer Institute. All patients gave informed consent. A total of 1662 patients were included.

Tissue microarrays (TMAs) were constructed from the 739 patients for which tumour material was available and were stained for ERα, progesterone receptor (PR), HER2 and the various PI3K/MAPK proteins. ER and PR were considered positive if ≥10% of tumour cells showed nuclear staining. HER2 was considered positive if membranous staining was DAKO score 3. If the DAKO score was 2, chromogenic in situ hybridization was performed and considered positive if the sample showed HER2 amplification. Immunohistochemistry for Ki67 was performed using the monoclonal mouse anti-human Ki67 antigen clone MIB-1 (DAKO, Agilent Technologies, Santa Clara, California, USA) and a standard staining protocol on the Ventana Benchmark® Ultra system (Ventana Medical Systems, Tucson, USA). The proportion of invasive tumour cells with nuclear staining was assessed by MO, a cut-off for binary score of <10% *vs* ≥10% was used in the analyses. During the scoring of the PI3K/MAPK proteins, cases with ductal carcinoma *in situ* or without residual invasive tumour and cores with technical errors were excluded. The number of tumour samples with staining results for all proteins was further diminished due to competing studies requiring slides from the same TMAs.

## Supplementary Results

### *Expression of p-4EBP1 and p-S6RP, tamoxifen benefit and prognosis in ER-positive patients*

In the group of ER-positive patients (N = 563), a total of 132 RFI events occurred. The median follow-up of patients without a recurrence event was 8.2 years. There were 430 and 442 ER-positive breast cancer cases that could be evaluated for p-4EBP1 and p-S6RP expression, respectively.

After evaluating multiple cut-off points in patients evaluable for staining results, we found that dichotomization of ≤20% *vs* >20% for p-4EBP1 and of negative *vs* positive for p-S6RP provided the best cut-off. Tumours with low and high p-4EBP1 (Table S2) and those with absent or present p-S6RP (Table S2) were analysed for a possible association with a series of patient and tumour characteristics. Both proteins were significantly associated with the other six proteins of the PI3K and/or MAPK pathways. Tumours with a positive PR preferentially stained high for p-4EBP1 (p-4EBP1 high 66% and low 34%), which was also the case for tumours with a lower grade (p-4EBP1 high 64% and low 36%). Tumours with a positive PR were preferentially positive for p-S6RP (p-S6RP positive 68% and negative 32%), while those negative for p-S6RP were preferentially of lower grade (grade 1-2 70% and grade 3 30%). Patients with tumours harbouring high levels of p-4EBP1 experienced benefit from adjuvant tamoxifen (multivariate Hazard Ratio (HR) 0.39, 95% Confidence Interval (CI) 0.20 – 0.76, *p* = 0.006), while those with low tumour levels of p-4EBP1 did not (multivariate HR 0.74, 95% CI 0.32 – 1.74, *p* = 0.49) (Figures S2A and S2B). Patients with tumours without p-S6RP expression derived significant tamoxifen benefit (multivariate HR 0.28, 95% CI 0.12 – 0.67, *p* = 0.004), while those with positive tumour staining had no benefit (multivariate HR 0.64, 95% CI 0.33 – 1.21, *p* = 0.17) (Figure S2C and S2D). The tests for interaction between p-4EBP1 or p-S6RP and treatment were not significant (*p* = 0.23 and *p* = 0.12, respectively) indicating no significant difference in tamoxifen benefit according to the protein subgroups.

We explored tamoxifen efficacy in lymph node-positive and lymph node-negative patients separately. As expected from the interim analyses in the original trial, all lymph node-positive patients derived significant benefit from tamoxifen regardless of various p-4EBP1 or p-S6RP cut-off points. However, dichotomization of both p-4EBP1 and p-S6RP by ≤10% vs >10% in lymph node-negative patients revealed differences in tamoxifen outcome. In the Cox models, the test for interaction between treatment arm and protein expression was significant, although only in the univariate analysis (p-4EBP1: univariate *p* = 0.049 and multivariate *p* = 0.10; p-S6RP: univariate *p* = 0.016 and multivariate *p* = 0.075). Patients with low tumour levels of p-4EBP1 had no tamoxifen benefit (multivariate HR 2.67, 95% CI 0.70 – 10.3, *p* = 0.15), while in patients with high levels more benefit was seen (multivariate HR 0.64, 95% CI 0.22 – 1.88, *p* = 0.42). On the contrary, a tamoxifen benefit was observed in patients with p-S6RP tumour levels ≤10% (multivariate HR 0.45, 95% CI 0.14 – 1.43, *p* = 0.18), while no benefit was observed in patients with p-S6RP levels of >10% (multivariate HR 2.17, 95% CI 0.60 – 7.87, *p* = 0.24).

The prognostic potential of high *vs* low p-4EBP1 and positive *vs* negative p-S6RP was analysed only in patients randomized to the control arm to rule out bias from treatment. There was no significant association of either p-4EBP1 or p-S6RP expression with prognosis (multivariate HR = 1.47, 95% CI 0.54 – 3.97, *p =* 0.45 and multivariate HR = 0.59, 95% CI 0.23 – 1.57, *p =* 0.24, respectively).

## Supplementary tables

**Table S1**. Antibodies used for immunohistochemistry, scoring procedures and kappa coefficients to determine the interobserver variability

| **Antibody** | **Art. No. (clone)^1^** | **Scoring system** | **Scoring for hierarchical clustering** | **Comparable cores from N patients for kappa calculation** | **Cut-off for binary score for kappa calculation** | **Kappa for binary score** |
| --- | --- | --- | --- | --- | --- | --- |
| PTEN | 9559 (138G6) | cytoplasmic intensity | 0 – 3 | 126 | 0 *vs* 1-3 | 0.75 |
| p-AKT (Thr308) | 2965 (C31E5E) | cytoplasmic intensity | 0 – 3 | 133 | 0 *vs* 1-3 | 0.53 |
| p-AKT (Ser473) | 4060 (D9E) | cytoplasmic intensity | 0 – 3 | 94 | 0-1 *vs* 2-3 | 0.70 |
| p-4EBP1 (Ser65) | 9456 (174A9) | percentage of tumour cells with nuclear staining | 0 – 100% | 112 | 0-50 *vs* 51-100 | 0.59 |
| p-p70S6K (Thr389) | 9206 (1A5) | cytoplasmic intensity | 0 – 3 | 97 | 0 *vs* 1-3 | 0.44 |
| p-ERK1/2 (Thr202/Tyr204) | 4370 (D13.14.4E) | proportion of tumour cells with nuclear staining | 0 – 100% | 69 | negative *vs* positive | 0.85 |
| p-S6RP (Ser235/236) | 2211 | percentage of tumour cells with cytoplasmic and membranous staining | 0 – 100% | 85 | 0-19 *vs* 20-100 | 0.55 |

^1^all from Cell Signalling Technology, Danvers, MA, US

**Table S2**. Association between clinico-pathological factors and downstream proteins in low vs high p-4EBP1 and negative vs positive p-S6RP

|  |  |  | p-4EBP1 (N = 430) |  |  | p-S6RP (N = 442) |  |
| --- | --- | --- | --- | --- | --- | --- | --- |
|  |  | ≤20% | >20% |  | Negative (161) | Positive (281) |  |
|  |  | N (%) | N (%) | *p*-value* | N (%) | N (%) | *p*-value* |
| Age | <65 | 72 (42%) | 130 (50%) | 0.11 | 72 (45) | 138 (49) | 0.43 |
|  | ≥65 | 99 (58%) | 129 (50%) |  | 89 (55) | 143 (51) |  |
| Lymph node status | Negative | 94 (55%) | 138 (53%) | 0.77 | 86 (53) | 151 (54) | 1 |
|  | Positive | 77 (45%) | 121 (47%) |  | 75 (47) | 130 (46) |  |
| T stage | T1-2 | 145 (85%) | 234 (90%) | 0.09 | 139 (86) | 252 (90) | 0.35 |
|  | T3-4 | 26 (15%) | 25 (10%) |  | 22 (14) | 29 (10) |  |
| Grade | Grade 1-2 | 97 (57%) | 176 (68%) | 0.019 | 113 (70) | 167 (59) | 0.025 |
|  | Grade 3 | 74 (43%) | 83 (32%) |  | 48 (30) | 114 (41) |  |
| Histological subtype | Ductal | 132 (89%) | 193 (92%) | 0.46 | 119 (88) | 211 (92) | 0.2 |
|  | Lobular | 16 (11%) | 17 (8%) |  | 17 (12) | 18 (8) |  |
| HER2 status | Negative | 149 (89%) | 234 (93%) | 0.11 | 148 (95) | 243 (89) | 0.05 |
|  | Positive | 19 (11%) | 17 (7%) |  | 8 (5) | 30 (11) |  |
| PR status | Negative | 97 (57%) | 114 (44%) | 0.01 | 89 (56) | 128 (46) | 0.047 |
|  | Positive | 73 (43%) | 144 (56%) |  | 70 (44) | 151 (54) |  |
| PTEN | Negative | 51 (32%) | 20 (9%) | <0.0001 | 40 (29) | 34 (13) | 0.00023 |
|  | Positive | 108 (68%) | 212 (91%) |  | 99 (71) | 225 (87) |  |
| p-AKT(Thr308) | 0 | 114 (68%) | 120 (48%) | <0.0001 | 107 (71) | 132 (49) | <0.0001 |
|  | 1-3 | 53 (32%) | 129 (52%) |  | 43 (29) | 140 (51) |  |
| p-AKT(Thr473) | 0-1 | 91 (63%) | 65 (30%) | <0.0001 | 73 (60) | 87 (36) | <0.0001 |
|  | 2-3 | 54 (37%) | 153 (70%) |  | 49 (40) | 158 (64) |  |
| p-p70S6K | 0 | 91 (56%) | 77 (32%) | <0.0001 | 86 (61) | 86 (32) | <0.0001 |
|  | 1-3 | 71 (44%) | 163 (68%) |  | 55 (39) | 180 (68) |  |
| p-S6RP | Negative | 76 (46%) | 71 (29%) | 0.0008 | NA | NA |  |
|  | Positive | 91 (54%) | 173 (71%) |  | NA | NA |  |
| p-4EBP1 | ≤20% | NA | NA |  | 76 (52) | 91 (34) | 0.0008 |
|  | >20% | NA | NA |  | 71 (48) | 173 (66) |  |
| p-ERK1/2 | Negative | 100 (62%) | 72 (29%) | <0.0001 | 97 (67) | 79 (30) | <0.0001 |
|  | Positive | 62 (38%) | 174 (71%) |  | 47 (33) | 186 (70) |  |

* Fisher’s exact test based on cases without missing values

**Table S3A**. Univariate Cox proportional hazard model of recurrence free interval (RFI) including heatmap group and treatment interaction, stratified for lymph node status.

| **Variable** |  | **HR** | **95% CI** | ***p*** |
| --- | --- | --- | --- | --- |
| Interaction | Cluster vs treatment |  |  | 0.007 |
| Tamoxifen vs CON (ref) | In cluster N | 0.23 | 0.12 – 0.44 | 0.0000081 |
|  | In cluster A | 1.53 | 0.44 – 5.25 | 0.50 |

**Table S3B.** Multivariate Cox proportional hazard model for prognosis, stratified for lymph node status, including heatmap group in control patients only

| **Variable** |  | **HR** | **95% CI** | ***p*** |
| --- | --- | --- | --- | --- |
| Cluster A vs N (ref) | CON patients | 0.061 | 0.0079 – 0.47 | 0.0073 |
| Age | ≥65 vs <65 (ref) | 0.80 | 0.27 – 2.4 | 0.69 |
| T stage | T3-4 vs T1-2 (ref) | 2.0 | 0.44 – 9.5 | 0.37 |
| Grade | Grade 3 vs grade 1-2 (ref) | 1.3 | 0.12 – 14 | 0.82 |
| PR status | Positive vs negative (ref) | 2.0 | 0.66 – 6.1 | 0.22 |
| Histological subtype | Lobular vs ductal (ref) | 5.3 | 0.75 – 38 | 0.095 |
| Ki67 | ≥10% vs <10% (ref) | 5.9 | 0.58 – 60 | 0.13 |
| Mitotic count | ≥8 / 2mm² vs <8 / 2mm² (ref) | 0.26 | 0.027 – 2.5 | 0.24 |

**Table S3C.** Multivariate Cox proportional hazard model of recurrence free interval (RFI) including classification tool groups and treatment interaction

| **Variable** |  | **HR** | **95% CI** | ***p*** |
| --- | --- | --- | --- | --- |
| Interaction | Cluster vs treatment |  |  | 0.024 |
| Tamoxifen vs CON (ref) | In rule FALSE | 0.24 | 0.11 – 0.49 | 0.00012 |
|  | In rule TRUE | 1.3 | 0.36 – 4.7 | 0.69 |
| Rule TRUE vs FALSE (ref) | CON patients | 0.24 | 0.066 – 0.91 | 0.036 |
|  | TAM patients | 1.3 | 0.64 – 2.8 | 0.43 |
| Age | ≥65 vs <65 (ref) | 1.0 | 0.59 – 1.8 | 0.89 |
| T stage | T3-4 vs T1-2 (ref) | 2.0 | 0.92 – 4.2 | 0.082 |
| Grade | Grade 3 vs grade 1-2 (ref) | 1.9 | 0.81 – 4.6 | 0.14 |
| PR status | Positive vs negative (ref) | 1.4 | 0.79 – 2.5 | 0.24 |
| Histological subtype | Lobular vs ductal (ref) | 2.7 | 0.99 – 7.5 | 0.053 |
| Ki67 | ≥10% vs <10% (ref) | 1.2 | 0.56 – 2.5 | 0.65 |
| Mitotic count | ≥8 / 2mm² vs <8 / 2mm² (ref) | 0.72 | 0.29 – 1.8 | 0.47 |

**Table S3D.** Univariate Cox proportional hazard model of recurrence free interval (RFI) including classification tool groups and treatment interaction

| **Variable** |  | **HR** | **95% CI** | ***p*** |
| --- | --- | --- | --- | --- |
| Interaction | Cluster vs treatment |  |  | 0.004 |
| Tamoxifen vs CON (ref) | In rule FALSE | 0.21 | 0.11 – 0.41 | 0.0000050 |
|  | In rule TRUE | 1.6 | 0.47 – 5.4 | 0.46 |

**Table S3E.** Multivariate Cox proportional hazard model for prognosis including classification tool groups in control patients only

| **Variable** |  | **HR** | **95% CI** | ***p*** |
| --- | --- | --- | --- | --- |
| Rule TRUE vs FALSE (ref) | CON patients | 0.065 | 0.0091 – 0.47 | 0.0067 |
| Age | ≥65 vs <65 (ref) | 0.71 | 0.23 – 2.2 | 0.55 |
| T stage | T3-4 vs T1-2 (ref) | 3.1 | 0.57 – 17 | 0.19 |
| Grade | Grade 3 vs grade 1-2 (ref) | 1.2 | 0.11 – 13 | 0.88 |
| PR status | Positive vs negative (ref) | 1.8 | 0.60 – 5.3 | 0.30 |
| Histological subtype | Lobular vs ductal (ref) | 4.6 | 0.62 – 33 | 0.14 |
| Ki67 | ≥10% vs <10% (ref) | 6.0 | 0.61 – 58 | 0.12 |
| Mitotic count | ≥8 / 2mm² vs <8 / 2mm² (ref) | 0.25 | 0.026 – 2.4 | 0.23 |

## Supplementary figures

**
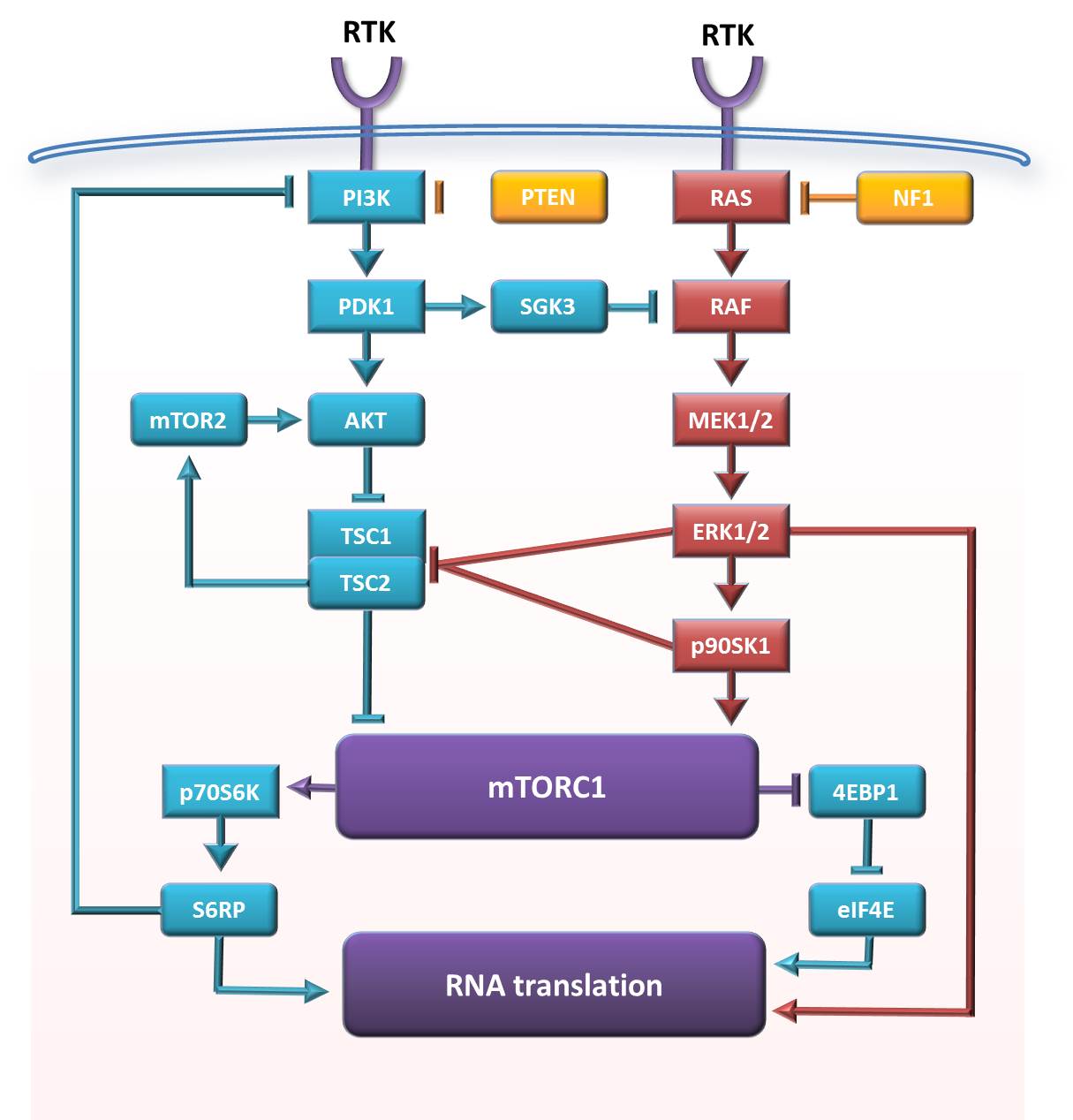
**

**Figure S1.** Canonical phosphatidylinositol 3-kinase (PI3K) (in blue) and mitogen-activated protein kinase (MAPK) (in red) signalling network, showing examples of cross-talk and feedback loops. Both pathways function downstream of receptor tyrosine kinases (RTKs) and G protein-coupled receptors. When PI3K is activated, it triggers a cascade of downstream proteins. PI3K is responsible for the phosphorylation of phosphatidylinositol diphosphate (PIP2) to phosphatidylinositol triphosphate (PIP3), which in turn activates AKT. AKT activates mammalian target of rapamycin complex (mTOR) complex 1 (mTORC1) by inhibiting the tuberous sclerosis (TSC) complex (TSC1 combined with TSC2) (1). Activation of mTORC1 phosphorylates p70 ribosomal protein S6 kinase (p70S6K) and eukaryotic translation initiation factor eukaryotic initiation factor 4E (eIF4E)-binding protein 1 (4EBP1) (2). When S6K is activated, it can phosphorylate 40S ribosomal protein S6 (S6RP), while phosphorylation of 4EBP1 leads to its dissociation from eIF4E, both processes initiating RNA translation (3). Phosphatase and tensin homolog (PTEN) is known as a tumour suppressor of the PI3K pathway as it directly dephosphorylates PIP3 to PIP2. When an RTK or G protein-coupled receptor becomes activated, it is also able to activate the MAPK pathway by phosphorylating RAS. Phosphorylated RAS activates RAF which leads to the activation of mitogen-activated and extracellular signal-regulated kinase kinase (MEK)1/2. Activation of MEK 1/2 in turn can phosphorylate extracellular signal-regulated kinase (ERK)1/2 (1). Phosphorylated ERK1/2 has many targets, including p90 ribosomal six kinase-1 (p90RSK1), which leads to cross-talk with the PI3K pathway through mTORC1, S6K, S6RP and eIF4B (4). However, phosphorylated ERK1/2 can also translocate to the nucleus to influence gene expression and regulate various transcription factors by itself (5). Neurofibromatosis type 1 (NF1) deactivates RAS and therefore functions as a tumour suppressor for this pathway (6).

Other abbreviations: PDK1: phosphoinositide-dependent protein kinase 1; SGK3: serum- and glucocorticoid-regulated kinase 3;


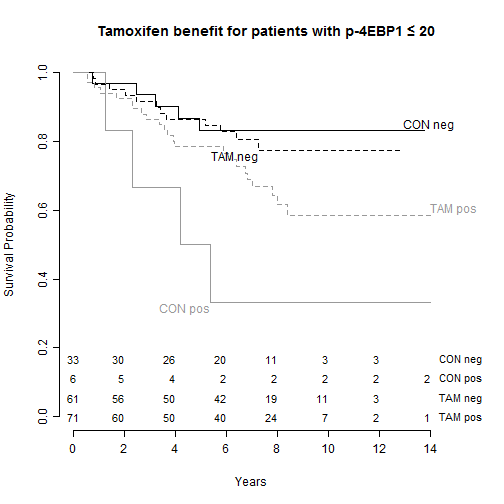
**
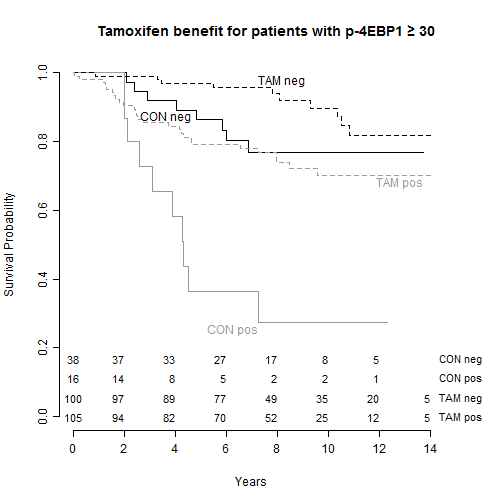
**
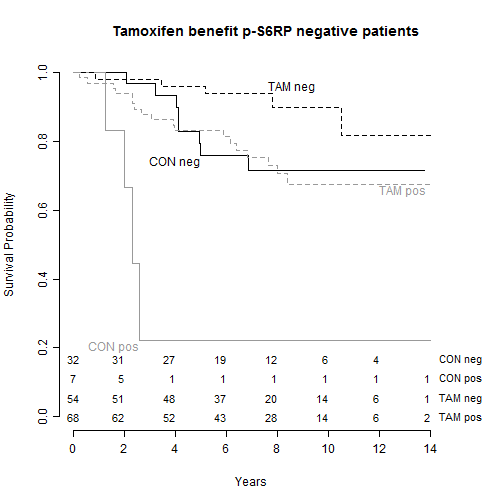
**
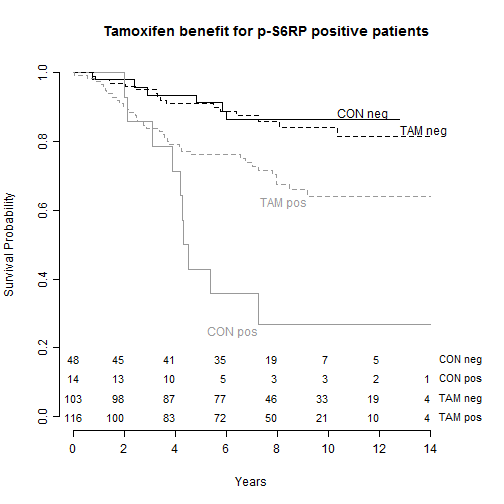
**

D

C

B

A

**Figure S2**. Kaplan-Meier curves for recurrence-free interval according to tamoxifen treatment in patients with low expression of p-4EBP1 in tumour tissue (A), high expression of p-4EPB1 (B), no expression of p-S6RP (C) and any expression of p-S6RP (D). The interrupted line specifies patients treated with tamoxifen. The continuous line shows patients randomized to the control arm. The light grey line specifies patients with lymph node-positive disease. The black line shows patients with lymph node-negative disease.

## Supplementary references

1. Saini KS, Loi S, de Azambuja E, Metzger-Filho O, Saini ML, Ignatiadis M, et al. Targeting the PI3K/AKT/mTOR and Raf/MEK/ERK pathways in the treatment of breast cancer. Cancer treatment reviews. 2013;39(8):935-46.

2. Dancey J. mTOR signaling and drug development in cancer. Nature reviews Clinical oncology. 2010;7(4):209-19.

3. Li S, Kong Y, Si L, Chi Z, Cui C, Sheng X, et al. Phosphorylation of mTOR and S6RP predicts the efficacy of everolimus in patients with metastatic renal cell carcinoma. BMC cancer. 2014;14:376.

4. McCubrey JA, Steelman LS, Chappell WH, Abrams SL, Montalto G, Cervello M, et al. Mutations and deregulation of Ras/Raf/MEK/ERK and PI3K/PTEN/Akt/mTOR cascades which alter therapy response. Oncotarget. 2012;3(9):954-87.

5. Asati V, Mahapatra DK, Bharti SK. PI3K/Akt/mTOR and Ras/Raf/MEK/ERK signaling pathways inhibitors as anticancer agents: Structural and pharmacological perspectives. European journal of medicinal chemistry. 2016;109:314-41.

6. Kourea HP, Zolota V, Scopa CD. Targeted pathways in breast cancer: molecular and protein markers guiding therapeutic decisions. Current molecular pharmacology. 2014;7(1):4-21.
